# Tumor-Specific Growth Factor (TSGF): A Futuristic Tumor Biomarker in Early Diagnosis of Cancer

**DOI:** 10.34172/apb.2023.051

**Published:** 2022-04-30

**Authors:** Bhagyalakshmi Nair, Anisha Kuriakose, Bilha Baby, Lekshmi.R. Nath

**Affiliations:** Department of Pharmacognosy, Amrita School of Pharmacy, Amrita VishwaVidyapeetham, AIMS Health Science Campus, Ponekkara P.O., Kochi, Kerala 682041, India.

**Keywords:** Biomarker, Cancer, Clinical implications, Diagnosis, Tumor-Specific growth factor

## Abstract

Despite the significant improvement in the treatment modalities, cancer is one of the fastest-growing chronic disease conditions all over the world. Genetic and Epigenetic alterations in the normal physiology of the cell are the key factor for tumor development. These changes can trigger the production of abnormal protein expressions through stimulation of different signaling pathways and can deeply affect normal cell growth and proliferation. Any altered protein expression, genetic variation, micro-RNA or post-translational protein modifications that indicate tumorigenesis can act as an early signal termed as biomarker. Cancer, being a multistep process with accumulating genetic and epigenetic alterations, could be detected early with suitable biomarkers. There are several proteins such as AFP, CA-125, PSA, troponin, CEA, osteopontin, CA 19-9 that act as biomarkers which help in early detection, prognosis, and monitoring of disease progression, a hunt for newer biomarkers with higher specificity and sensitivity is still ongoing. Tumor-specific growth factor (TSGF) is one such budding and prevailing tumor biomarker used for the early-stage detection of several types of carcinomas. TSGF is a gene that helps in tumor angiogenesis and gets released during the preliminary stages from cancer cells that ensure the vascular proliferation of the same. In this review, the clinical investigations of TSGF in different kinds of malignancy is discussed in detail and suggests the possibility of using TSGF as a biomarker in early diagnosis of cancer.

## Introduction

 Cancer is a significant public health concern that affects a wide range of populations globally. According to the reports published in 2021, about 1 898 160 new cancer cases and 608 570 cancer deaths were estimated worldwide^1^ with lung, breast, and prostate carcinomas as the most frequent types.^2^ Carcinogenesis transforms normal cells into neoplastic cells due to damage or alterations in the genetic apparatus. This could be due to accumulating mutations, alterations in genetic expression, activation of tumor promotor gene, or inactivation of tumor suppressor gene.^3^ Several pathological processes, mainly inflammation, accompany tumor development. Chronic inflammatory mediators and growth factors are released by the cancer cells, disrupting the immune system due to alterations in the functions of various immune cells.^3-8^ Advanced technologies like anti-sense therapy, anti-cancer vaccines, viral/von-viral gene delivery systems, anti-gene therapy, and tumor suppressor gene therapy abolish genetic damage. However, their clinical application is limited and thus is a matter of future perception.^9^

 Regardless of the histological type, damage to genetic material and suppression of anti-tumor immunity are the common key factors that lead to cancer progression. After exposure to chemical, physical or biological carcinogens, local tissue damage leads to temporary suppression of the anti-tumor immunity for tissue repair. However, due to damages caused by exogenous factors, imbalance in the sympathetic/hyper-sympathetic dominance, or tissue hypoxia,^3,4^ the normal physiological process of tissue repair becomes a pathophysiological resulting in the formation of a cancer cell. Genetic and epigenetic variations cause altered protein expressions and also lead to carcinogenesis. Alteration in the protein expressions badly affects the cell physiology and metabolism, producing and transmitting signals to the neighboring cells.^10,11^

 A biomarker can be defined as a substance/ biomolecule that can be measured or as a structure or process that can be detected or predicted to analyze the outcome of several diseases.^12,13^ Cancer biomarkers constitute a wide range of biological entities such as nucleic acids, sugars, proteins, cytogenetic and cytokine entities, and neoplastic cells in circulating body fluids.^14^ These biochemical entities are produced by tumor cells that can be widely used for patient assessment in numerous clinical settings like estimating the risk of developing cancer, screening of primary occult neoplasm, the differential diagnosis between the benign and malignant tumors, predicting the response or progression to therapy and monitoring the recurrence of the disease condition.^15^ Cancer biomarker discovery is continuing as an active and productive area of research wherein clinicians and scientists are using the knowledge regarding tumors and the advent of novel technologies to develop a potential cancer biomarker. With the emergence of sophisticated and advanced genomic profiling techniques and targeted molecular therapies, identification and validation of tumor biomarkers is now a part of the cancer drug discovery process.^16^

**Figure 1:**
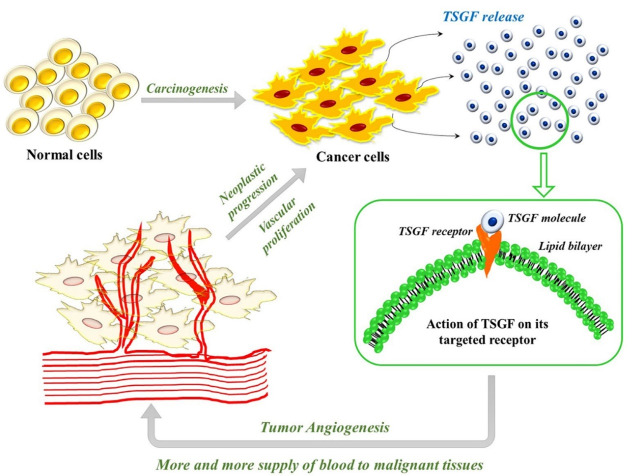
The above figure illustrates the role of tumor-specific growth factor (TSGF) in the early stages of carcinogenesis. TSGF is a highly specific and sensitive novel biomarker that helps n cancer cell proliferation and development. Mutation in the genetic apparatus of a cell triggers tumor development, and TSGF gets released from the cancerous cells that promote tumor angiogenesis and vascular proliferation, further worsening the condition

 Scholars first discovered tumor-specific growth factor (TSGF) at the University of Toronto in 1989. TSGF is an internationally recognized term for certain carbohydrates and metabolites like lipoproteins, amino acids, and enzymes that help grow and develop malignant cells.^17^ TSGFs are released by cancer cells during the early stages of tumor development and help in the proliferation of cells and peripheral blood capillaries. Thus, it promotes the angiogenesis of malignant cells, increasing the blood supply to cancer tissue.^17^ Scientific reports indicate no correlation between TSGF and non-malignant vascular proliferation and can be used as a potent indicator in distinguishing normal cells from neoplastic cells^17^ (Figure 1).Through this review, we aim to gather information on the studies conducted on the association of TSGF and various types of cancers and discuss the possibility of using TSGF as a potential biomarker for prediction/ early diagnosis/ detection/ prognosis and treatment outcome of each type.

## A layout of the clinical implications of TSGF in different types of cancer

 TSGF is produced by cancer cells and released into the bloodstream during the early stages of malignant tumor progression. The release of TSGF promotes the growth and proliferation of cancer cells and peripheral capillaries. Increased proliferation of peripheral blood capillaries facilitates more blood supply to tumor tissue which speeds up the tumor development^18,19^ The promotion of angiogenesis of tumor cells is through the cloned T lymphocytes differentiation by inhibiting the IgM and IgG production.^17^ The angiogenesis promoted with the help of TSGF produces high sensitivity and specificity with greater value in diagnosing malignant cells early.^20,21^ Thus, serum levels of TSGF can manifest the existence of cancer.

 Osteosarcoma is a progressive primary bone malignancy that commences from the immature stromal spindle cells. A study published in 2020 by Zhang et al reported that TSGF is a relatively specific serum marker for osteosarcoma. Seventy-five osteosarcoma patients were provided neoadjuvant chemotherapy with three different courses of treatment. Blood samples were collected before and after chemotherapy treatment and tested for serum markers like cyclooxygenase-2, vascular endothelial growth factor, transforming growth factor-beta, TSGF, and basic fibroblast growth factor. Serum levels of TSGF and other related tumor biomarkers were significantly decreased after chemotherapy treatment but were still higher than in the normal control group indicating that chemotherapy treatment can reduce the proliferation of osteosarcoma cells. The data from the retrospective study depicts that the mean serum levels of TSGF decreased significantly after chemotherapy. The serum TSGF levels of patients in an observational group before and after chemotherapy were 86.23±12.19 U/L and 52.86±8.41 U/L, respectively than that of the control group (25.5±4.95 U/L).^17^

 Breast cancer is one of the most common malignant tumors among the women population. Breast carcinoma badly influences women’s life and health and is the second leading cause of cancer mortality among women population across the globe. A retrospective study was performed to evaluate the clinical significance of Color Doppler ultrasound in combination with serum tumor biomarkers like TSGF, CEA, and CA15-3. Among 103 breast cancer patients, 50 patients were with benign breast lesions. Color Doppler Ultrasound revealed a substantial difference in terms of tumor morphology, tumor boundary, internal echo, peak blood flow velocity (Vmax), resistance index (RI), and pulsatility index (PI). The ultrasound images showcased irregular tumor shape with unclear tumor boundaries and increased Vmax, RI, and PI.^19^

 Subsequently, the serum levels of TSGF were drastically higher in patients with breast cancer (157.69±46.72 U/mL) compared with benign lesion subjects (50.24±15.61 U/mL). Also, the serum TSGF level was found to be declined after breast cancer treatment (107.82±32.97 U/mL) than those before treatment. From the combined detection, ROC curve analysis conveyed the area under the curve (AUC) of TSGF to be 0.843 while the Youden index to be 0.560. Also, the best cut-off value of TGSF was found to be 70.00 U/mL. Overall, the sensitivity and specificity of the combined detection color Doppler ultrasound and serum TSGF marker was 88.89%. The study demonstrates that the combined use of color Doppler ultrasound and serum markers can improve the diagnosis of breast cancer rather than using it as a single detection method.^19^

 Zhao et alconducted a similar study using 70 breast cancer patients to investigate the influence of neoadjuvant CAF chemotherapy on serum TSGF, CA 15-3, and CA-125 levels in breast cancer. All patients underwent surgery, but the observational group received neoadjuvant CAF chemotherapy (intravenous injection of cyclophosphamide, 5-fluorouracil, and adriamycin). It was tested for serum levels of tumor markers before and after treatment. Serum levels of TSGF and other cancer antigen markers were reduced after the treatment compared to pre-treatment. A positive correlation was also observed between TSGF and other cancer antigen markers, namely, CA 15-3 and CA-125. From the study reports, the overall response rate (ORR) and disease control rate (DCR) of the observational group was found to be 68.6% and 88.6%, respectively, when compared with the control group (ORR- 42.6% and DCR- 72.3%). Overall study results reveal that TSGF can be used as an effective biomarker in the effective evaluation of neoadjuvant CAF chemotherapy in breast carcinoma.^20^

 Endometrial cancer is the most common gynecological malignancy observed in perimenopausal and postmenopausal women, with a high mortality rate. Patients are more likely to be cured if endometrial cancer is diagnosed early. Thus, proper diagnosis is critical. A study conducted by Yu et al estimated the clinical value of TSGF and CA-125. Blood samples from peripheral veins were collected from all patients through a simple needle aspiration technique. Blood samples from uterine veins were collected from patients with endometrial cancer. The serum levels of TGSF and CA-125 from the blood samples collected through peripheral and uterine veins of endometrial cancer subjects were compared with those in uterine fibroids (uterine myoma) patients (non-tumor controls) and normal women subjects. In the disease group, the specificity and sensitivity of serum TSGF levels were 62.5% and 64.9%, respectively. Subsequently, the sensitivity of TSGF was gradually increased to 75.7% during the combined detection of TSGF and CA-125. The study data showed that the serum TSGF level was 72.2±13.3 U/mL in endometrial cancer patients compared to non-tumor patients (55.5±14.6 U/mL) and normal subjects (46.1±10.2 U/mL). It was found that the TSGF level was gradually increasing with the progression of endometrial carcinoma. Thus detection of serum TSGF biomarker in the blood can be valuable in the early diagnosis of endometrial malignancy.^21^

 In an alternative study by Chenet al, in 106 patients with endometrial carcinoma, the patients were divided into observation and control groups. Fifty-seven patients in the observational group had undergone laparoscopic surgery along with two doses of TC chemotherapy (paclitaxel + carboplatin). The serum TSGF levels before treatment were not significantly different between the observational and control group. But Chen et al reported changes in serum TSGF after TC chemotherapy and surgical treatment. The study results showed a drastic increase in the serum TSGF levels in control (88.72±12.67 U/L) and observational (89.66±13.18 U/L) groups before treatment. Whereas, after treatment, the serum TSGF was significantly reduced in the control (57.42±8.17 U/L) and observation (44.45±4.62 U/L) groups. Thus, the authors suggest TSGF molecule can be used to analyze the efficacy of adjuvant chemotherapy and related surgical treatment modalities.^22^

 oral squamous cell carcinoma (OSCC) is the most common but fatal type of Oral malignancy characterized by invasive growth and frequent regional metastasis. A case-controlled study was designed using 80 OSCC patients to investigate the use of three potential tumor biomarkers: long non-coding RNAs, TSGF and SCCA. From the study results, Shao et al showed that the serum TSGF levels were significantly high in different stages of tumor development with or without lymphatic metastasis. Also, the serum TSGF levels can distinguish between the OSCC and control subjects with an AUC of 0.648, a sensitivity of 63.3%, and a specificity of 66.7%.^23^

 In another study, Jie et al analyzed the therapeutic effect of doses of ^125^ I (a radioisotope of iodine) radioactive particle brachytherapy using serum TSGF and other tumor biomarkers in oral carcinoma. Out of the 78 oral carcinoma patients, one group received a high dose of ^125^ I radioactive particle brachytherapy, while the other group received a low dose of ^125^ I radioactive particle brachytherapy. It was found that a high dose of particle brachytherapy was more efficient in decreasing the serum levels of TSGF (59.73±6.12 µg/mL) than those of the low dose (68.53±7.12 µg/mL) group. The study demonstrates that high dose particle brachytherapy with radioactive ^125^ I is a safe and effective treatment in comparison with low-dose particle brachytherapy.^24^

 Pancreatic malignancy is the most common digestive malignancy with a high mortality rate, rapid proliferation, and diagnostic difficulties. A study was conducted to evaluate the efficacy of Cryoablation. An analysis was performed in 31 patients with pancreatic malignancy to assess the clinical significance of CA-242, CA 19-9, CA-125 (carbohydrate antigens), carcinoembryonic antigen (CEA), and TSGF before and after the cryoablation in pancreatic carcinoma. TSGF and other cancer antigens serum levels were measured before and one-month post-treatment. Serum levels of TSGF (17.0 ± 1.0 U/mL) were higher in the pancreatic cancer group than in the control group pre-treatment. But one-month post-treatment, the serum TSGF level (14.1 ± 0.9 U/mL) and other cancer antigen biomarkers were significantly reduced, thus making TSGF an essential index for the early detection and prognosis of pancreatic carcinoma.^25^

 Gastric carcinoma is yet another commonly observed malignancy with no obvious symptoms. But most of the patients clinically diagnosed with gastric malignancy will be already in the middle or advanced stage of tumor development, thus making them susceptible to delaying the best treatment regimen. A study conducted by Yin et al among gastric cancer patients (group 1) and benign gastric disease (group 2), along with a normal control group, estimated serum levels of TSGF and other tumor biomarkers. The study involved 40 patients with gastric carcinoma (GC), and the serum levels of the aforementioned tumor biomarkers were compared with the serum samples of 30 normal healthy volunteers and 40 patients with benign gastric diseases (GBD). From the study results, the serum levels of TSGF molecule in the GC group (76.19±11.84 U/mL) were found to be more than the GBD (62.27±11.45 U/mL) and healthy group (5.94±10.66 U/mL). Thus, the TSGF molecule can be used as an essential molecule for the early diagnosis and detection of gastric carcinoma.^26^

 Colon cancer is the most common alimentary canal malignancy with a higher mortality rate. To explore the expression and significance of TSGF along with CEA and AFP, a study was conducted by Hu et al on 43 colon cancer patients with a radical operation. The study reported that TSGF, CEA, and AFP were significantly high before radical surgery. One-month post-surgery, the expression rates of TSGF, CEA, and AFP drastically declined, thus suggesting the possibility of using TSGF as a biomarker to evaluate the effect of the radical operation on colon cancer. The results indicate elevated serum TSGF in subjects before radical surgery (77.33±7.02 U/mL) compared with the post-radical operation (72.14±6.13 U/mL). Thus, the author states that the TSGF molecule can be used as an effective tumor biomarker in evaluating Radical operation in colon cancer, tumor differentiation, and early diagnosis.^27^

 The mortality rate of rectal carcinoma is exponentially increasing due to accelerated life, eating disorders, and environmental factors. TSGF and many other tumor biomarkers are widely explored in different types of malignancy. In a study conducted on 100 patients with rectal carcinoma, Ji et al analyzed the specificity and sensitivity of serum TSGF using ELISA. The specificity of CEA, CA 15-3, TSGF, and CA-125 in the control and observation groups had significant differences. Also, the sensitivity of CEA, CA 15-3, TSGF, and CA-125 between the control and observation groups depicted substantial differences. The study. Results indicate that the sensitivity and specificity of TSGF were found to be 44.7% and 63.6%, respectively. Subsequently, the sensitivity of the combined detection of serum biomarkers was found to be 85.7%. Thus, the authors convey that the combined detection of tumor biomarkers was more sensitive than the single detection of different tumor biomarkers. The authors concluded that TSGF can be used for the early diagnosis of rectal cancer if appropriately validated and as a promising tumor biomarker in rectal malignancy diagnosis clinically.^28^

 The role of TSGF as a tumor biomarker in early detection and diagnosis has also been explored in non-small cell lung carcinoma (NSCLC). A retrospective analysis was conducted to determine the significance of TSGF along with other cancer antigen biomarkers like CEA, CYFRA 21-1 (Cytokeratin fragment antigens 21-1) and Neuron-specific enolases. The study demonstrated that the positive rate of each selected biomarker was observed at low levels during the NSCLC diagnosis. A significant serum TSGF was helpful for the primary diagnosis of NSCLC. The study found that the median and positive rates of TSGF were 56–67 μ/mL and 10.14%, and 4%, respectively. Even though the positive rate was lower, TSGF can be used as a budding tumor marker in diagnosing NSCLC. Depending on limited biomarker development, further validation is required for the determination of the specificity and sensitivity of TSGF in NSCLC patients.^29^

 Several reports suggest TSGF as an efficient diagnostic marker for detecting hepatocellular carcinoma since its sensitivity can reach 82% at the cut-off value of 62 U/L.^30-32^ A simultaneous determination of serum TSGF level and other tumor biomarkers, namely, AFP, CEA, TSA, and ferritin, showcased sensitivity and specificity of 98.4% and 99%, respectively, at the cut-off value of 65U/L^32^ (Table 1).

**Table 1:** The summary of clinical studies performed on tumor-specific growth factor (TSGF) with their published results

**Type of cancer**	**No. of patients **	**The outcome of the study**	**Reference**
Osteo-carcinoma	75 patients with osteocarcinoma 55 healthy patients	The serum level of TSGF was lowered after the successful chemotherapeutic regimen	^17^
Breast carcinoma	103 breast cancer patients 50 patients benign lesions	Combined use of Color Doppler Ultrasound with the detection of serum TSGF and other markers was found to be an effective tool in the early diagnosis of breast cancer	^19^
	70 patients	Neoadjuvant CAF therapy was found to be effective in breast cancer patients	^20^
Endometrial Cancer	37 patients	Combined detection of serum TSGF and CA-125 could be an effective approach for the early diagnosis of endometrial cancer.	^21^
	106 patients	TC chemotherapy significantly improved serum TSGF and other inflammatory markers in endometrial cancer patients	^22^
Oral Carcinoma	80 patients with OSCC 70 healthy subjects	TSGF, if validated through large-scale prospective study, can be a novel circulating biomarker for the detection of OSCC.	^23^
	78 patients	High-dose brachytherapy with radioactive ^125^ I exerted safe and effective treatment with clinical values more beneficial than the lower-dose treatment.	^24^
Pancreatic Cancer	31 pancreatic cancer patients	Combined detection of TSGF and other serum biomarkers was found to be effective for the early detection of pancreatic cancer and predicting the efficacy after cryoablation.	^25^
Gastric cancer	45 patients with gastric cancer 40 patients with benign gastric disease 30 healthy subjects	Combined detection of TSGF and other serum biomarkers depicted better sensitivity and accuracy than single detection.	^26^
Colon Cancer	43 patients	Serum TSGF can be used to evaluate the effect of radical surgery on the colon carcinoma	^27^
Rectal Cancer	100 patients with rectal carcinoma	Joint detection of serum TSGF and other biomarkers could improve the early diagnostic yield of rectal carcinoma	^28^
NSCLC	276 patients with NSCLC	A considerable amount of TSGF was found in the serum samples of NSCLC patients, with a significant positive rate of 10.14%	^29^
Hepatocellular Carcinoma	170 patients with HCC	Tumor marker determination with TSGF improved the positive rate of tumor diagnosis in Hepatocellular carcinoma	^32^

## Conclusion

 With tremendous knowledge of comprehensive genomic profiling and rapid changes in various molecular targeted therapies, a significant progress has been observed in cancer biology research. This ensures a good impact for the early detection, prognosis, diagnosis, and prevention of cancer. Despite such progression, cancer remains a deadly chronic disease with never-ending questions in the treatment modalities and screening methods. According to National Cancer Institute (NCI), a biomarker is a molecule that can be found in blood, body fluids, or tissue which can be used as a significant indication to detect any kind of disease ailment. TSGF is a tumor antigen marker that helps in the vascular proliferation of tumor cells, thereby promoting tumor angiogenesis. The correlation between TSGF and hyperplasia of neoplastic cells is reported in several studies, but no association was found between TSGF and non-cancerous cells. The release of highly sensitive TSGF molecule from malignant cells is triggered with increased blood flow during the early stage of cancer development which boosts tumor cell proliferation. Several studies demonstrate TSGF as a potential tumor biomarker in the early detection and diagnosis of malignancy in various cancer. All these studies established a positive correlation of TSGF molecule with other tumor-related cancer antigen biomarkers which is helpful in the prognosis and screening of cancer. To conclude, TSGF is a highly specific and sensitive tumor antigen marker that may be used alone or in combination for the early detection and diagnosis of several types of cancer. Proper validation through large-scale prospective research is required to ensure the more appropriate utility of serum TSGF as a biomarker molecule in the early cancer diagnosis.

## Acknowledgments

 We thank Dr Shanti Kumar Nair, Dean of Research, Amrita Vishwa Vidyapeetham and Dr Sabitha M, Principal, Amrita School of Pharmacy for providing the facilities to carry out the review work. We thank Dr Priya R Prabhu, Post-doctoral Research Associate, Fred Hutchinson Cancer Research Center, US for the language editing of the manuscript. The study was supported by Amrita Vishwa Vidyapeetham PG student Research fund.

## Competing Interests

 The authors declare no conflict of interest.

## Ethical Approval

 Not applicable.
